# Supplementary material for: Application of a novel haplotype‐based scan for local adaptation to study high‐altitude adaptation in rhesus macaques
Source: Evol Lett. 2021 May 22;5(4):408–21. doi: 10.1002/evl3.232 (PMC8327953; doi:10.1002/evl3.232)
Supplement: Supplementary file 3 — Table S3. Estimates of false positive rates for various demographic history normalization scenarios. [file EVL3-5-408-s007.docx]

**Table S3.** Estimates of false positive rates for various demographic history normalization scenarios.

| **Demographic History** | **Estimated False Positive Rate** |
| --- | --- |
| Matched | $9.908\times{10}^{-3}$ |
| “Rand” | $0$ |
| “Under” | $0$ |
| “Over” | $0.9538$ |
