# Supplementary material for: Application of a novel haplotype‐based scan for local adaptation to study high‐altitude adaptation in rhesus macaques
Source: Evol Lett. 2021 May 22;5(4):408–21. doi: 10.1002/evl3.232 (PMC8327953; doi:10.1002/evl3.232)
Supplement: Supplementary file 4 — Table S4. Bin boundaries (# of sites), number of windows per bin, and top 1% thresholds for the XP‐nSL analysis of rhesus macaques. [file EVL3-5-408-s010.docx]

**Table S4.** Bin boundaries (# of sites), number of windows per bin, and top 1% thresholds for the XP-nSL analysis of rhesus macaques.

| **Bin Boundaries (min # sites, max # sites)** | **# Windows in Bin** | **Top 1% Threshold (Fraction of Scores > 2)** |
| --- | --- | --- |
| $(11, 731)$ | $2672$ | $0.346755$ |
| $(732, 987)$ | $2680$ | $0.262945$ |
| $(988, 1155)$ | $2676$ | $0.207089$ |
| $(1156, 1285)$ | $2675$ | $0.196678$ |
| $(1286, 1390)$ | $2663$ | $0.226605$ |
| $(1391, 1484)$ | $2687$ | $0.19274$ |
| $(1485, 1576)$ | $2665$ | $0.163597$ |
| $(1577, 1681)$ | $2648$ | $0.169275$ |
| $(1682, 1824)$ | $2666$ | $0.164025$ |
| $(1825, 5766)$ | $2666$ | $0.153176$ |
