# Supplementary figures and images for: Application of a novel haplotype‐based scan for local adaptation to study high‐altitude adaptation in rhesus macaques
Source: Evol Lett. 2021 May 22;5(4):408–21. doi: 10.1002/evl3.232 (PMC8327953; doi:10.1002/evl3.232)

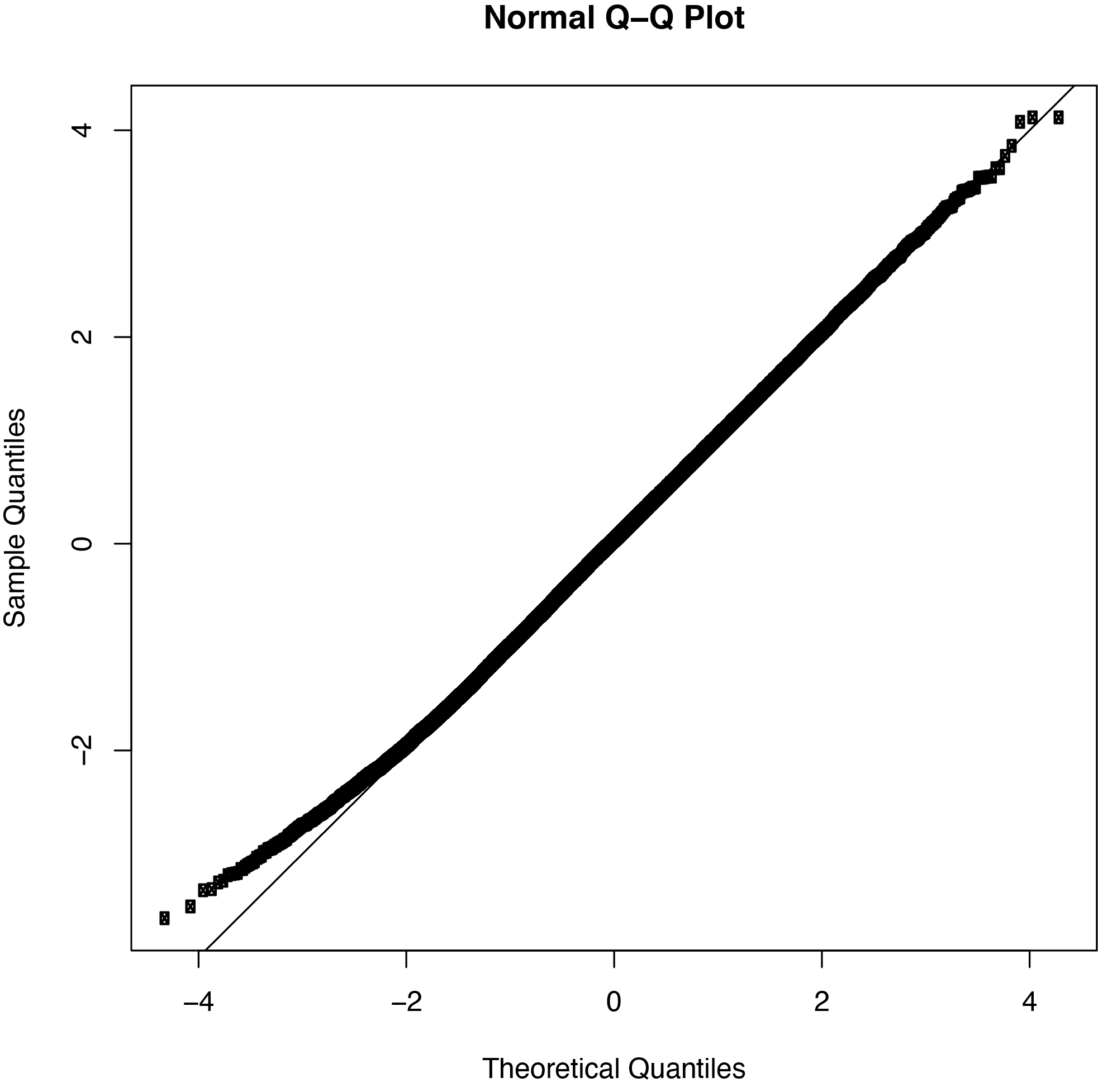

Supplement: Supplementary file 5 — Figure S1. A normal quantile‐quantile plot of neutral XP‐nSL scores showing generally good adherence to a standard normal distribution. Due to autocorrelation along the genome, only every 1000th score is plotted. [file EVL3-5-408-s003.png]

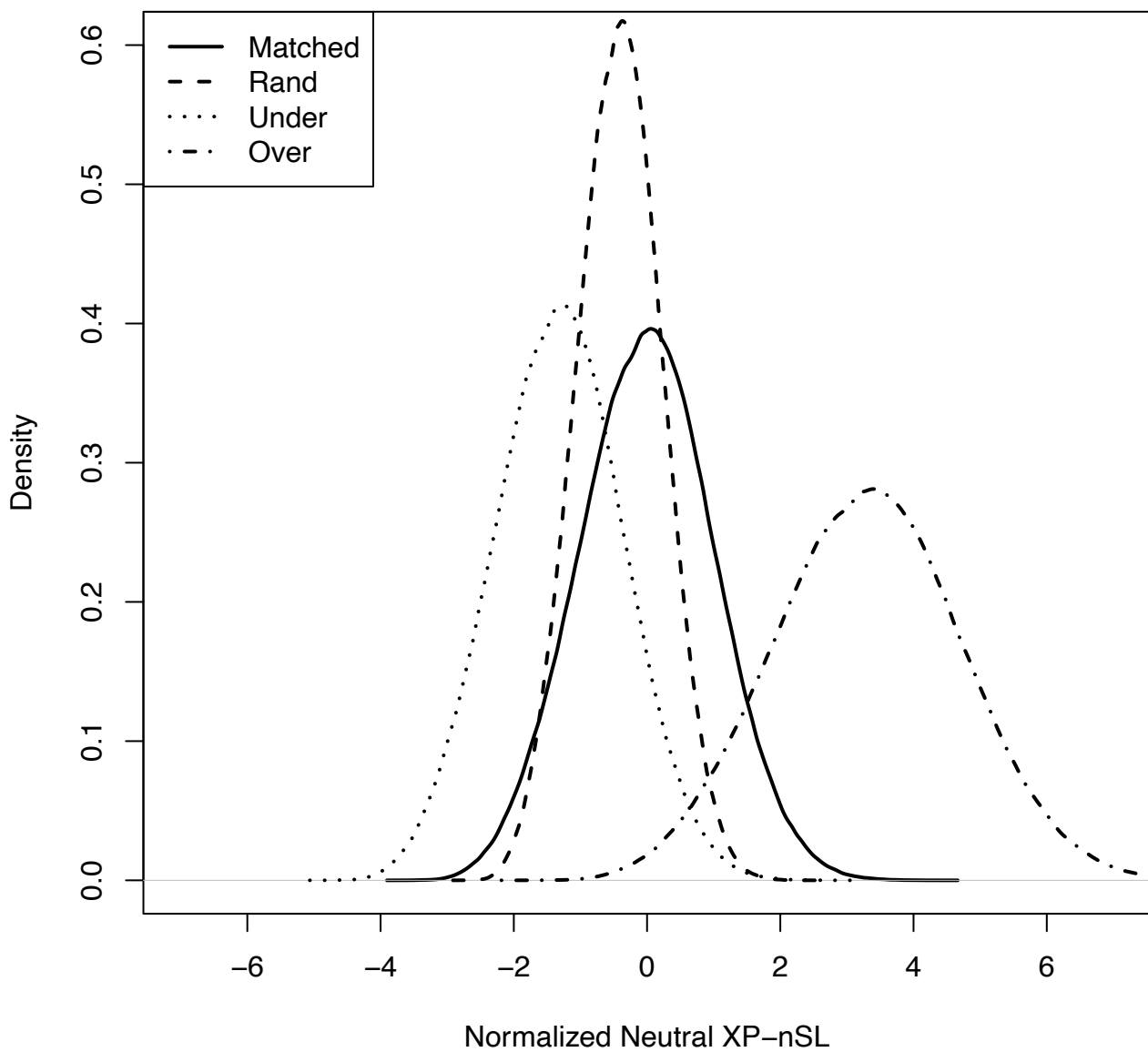

Supplement: Supplementary file 6 — Figure S2. The distribution of neutral XP‐nSL scores normalized with a matched demographic history (solid line), normalized with the “Rand” demographic history (dashed line), normalized with the “Under” demographic history (dotted line), and normalized with the “Over” demographic history (dash‐dot line). Normalizing with the wrong demographic history can dramatically shift the distribution of neutral XP‐nSL scores. [file EVL3-5-408-s011.pdf]

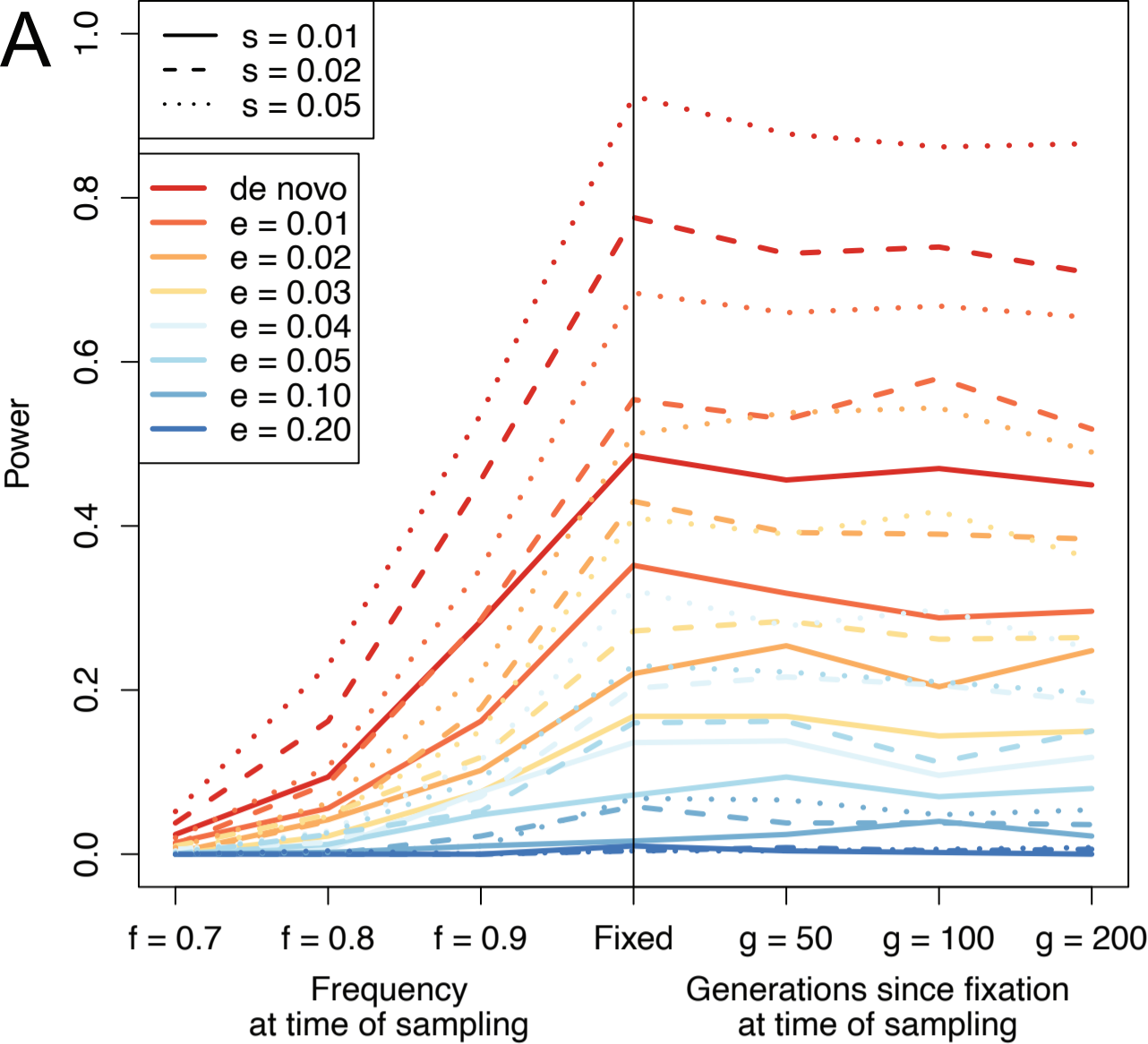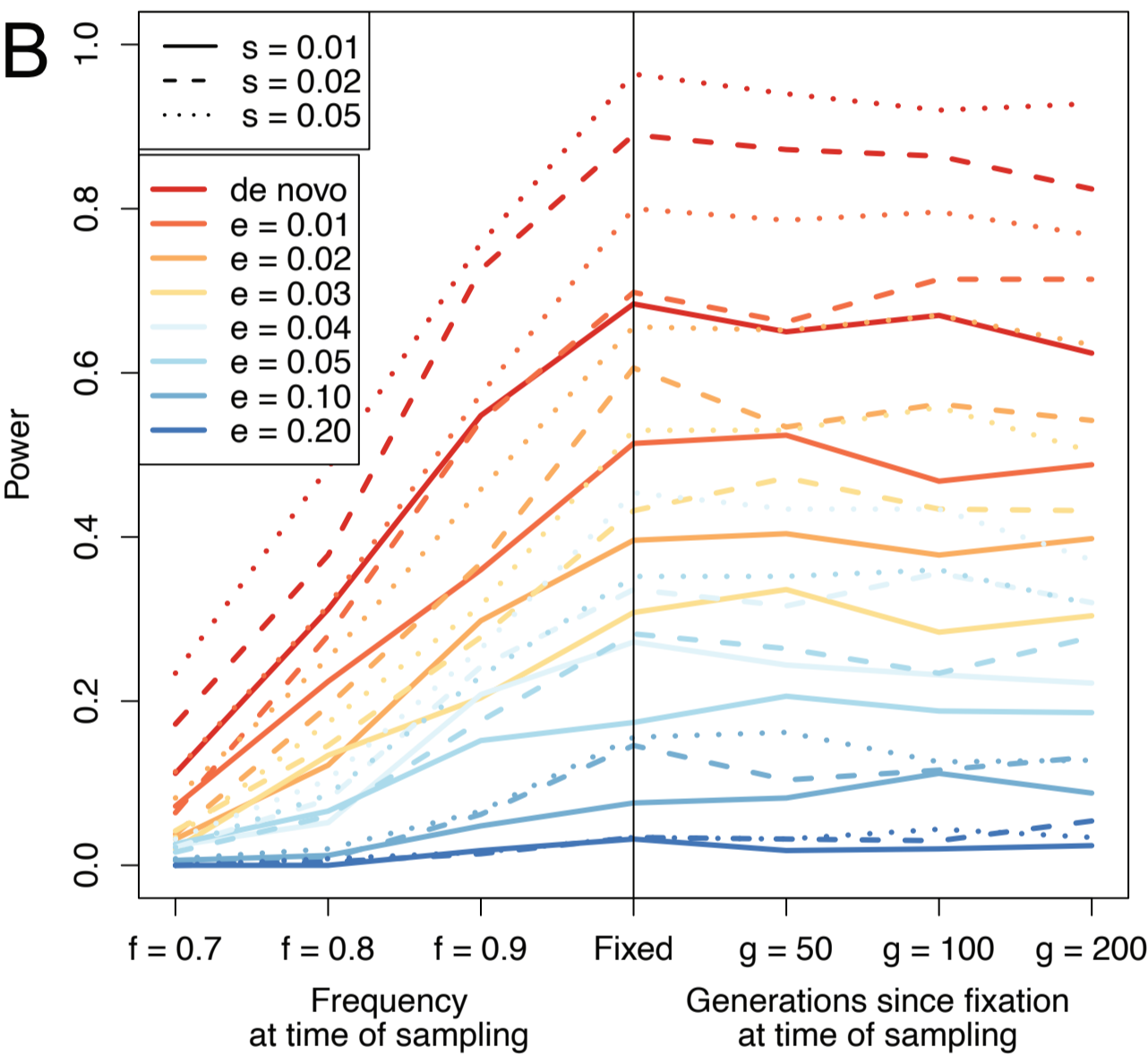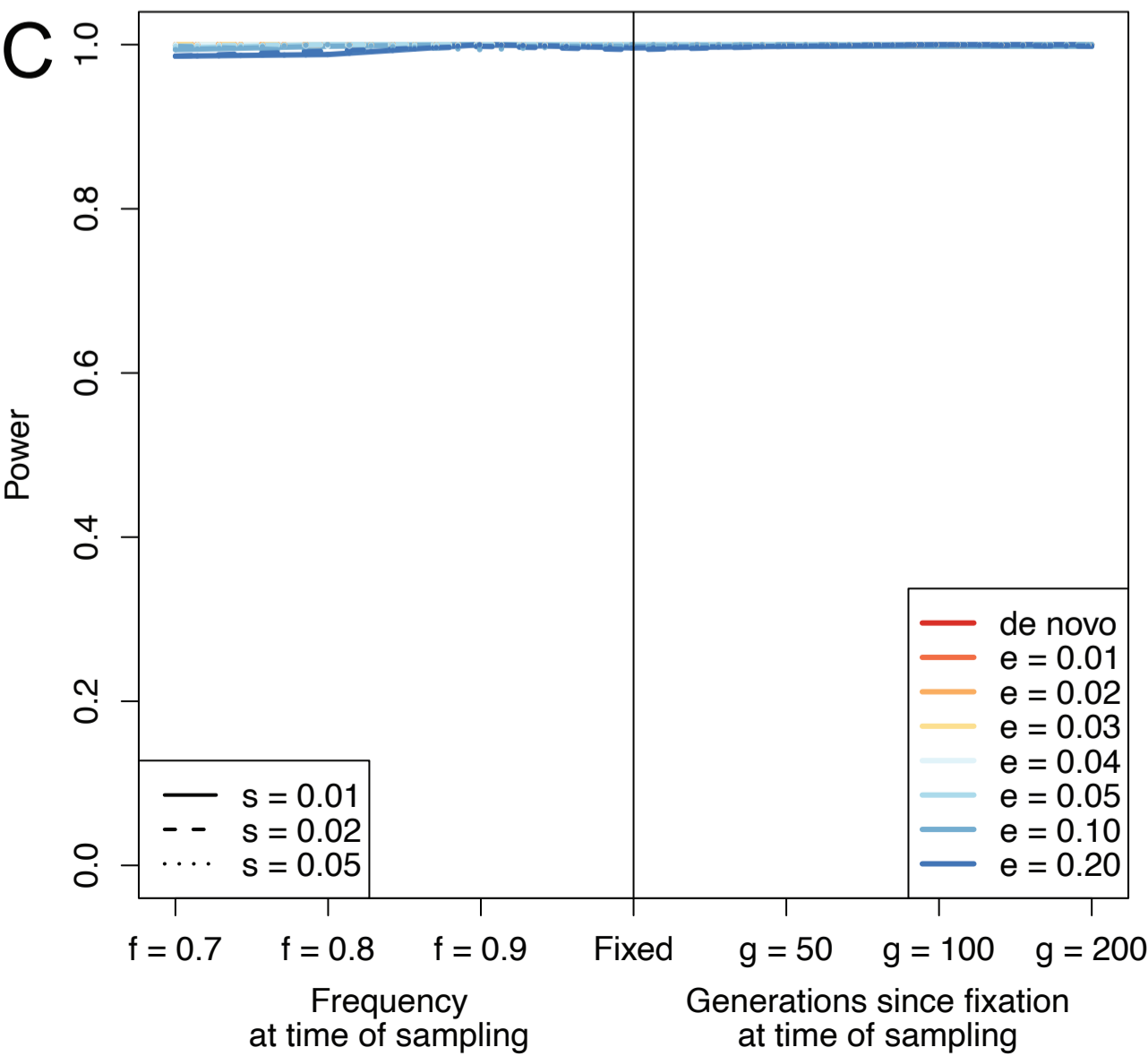

Supplement: Supplementary file 7 — Figure S3. XP‐nSL power using mismatched demographic histories for normalization. (A) Using the “Rand” history. (B) Using the “Under” history. (C) Using the “Over” history. [file EVL3-5-408-s005.pdf]

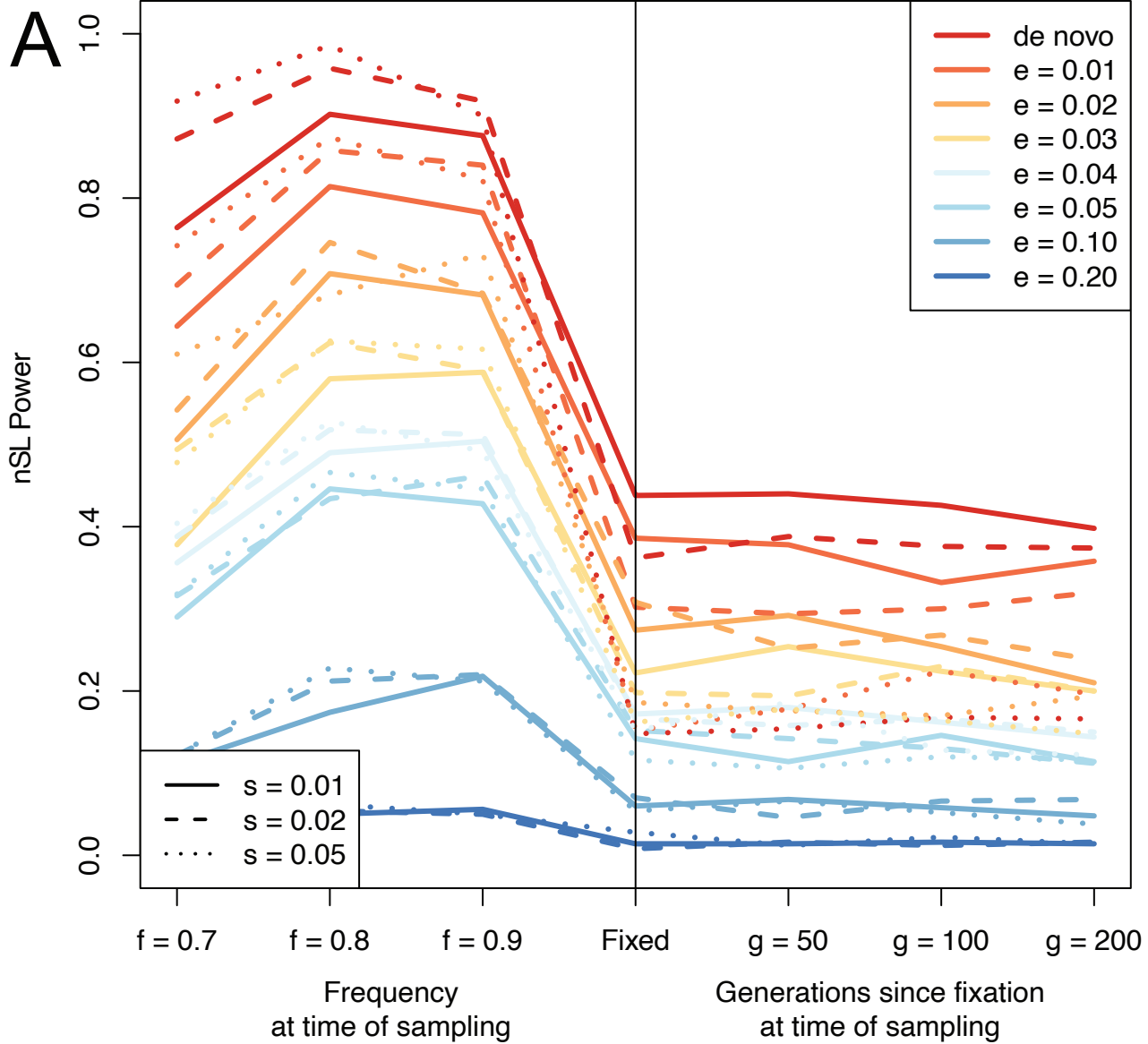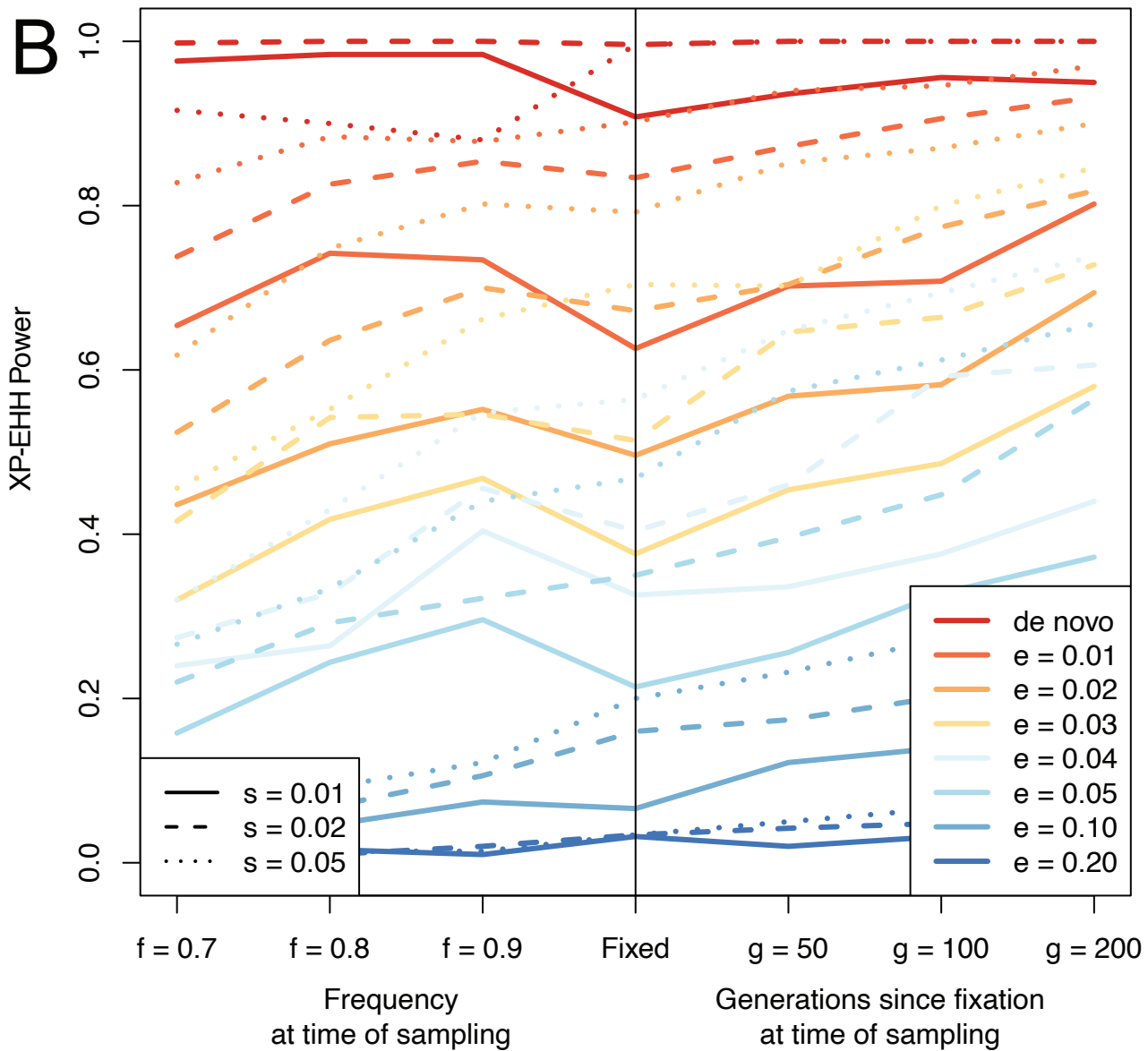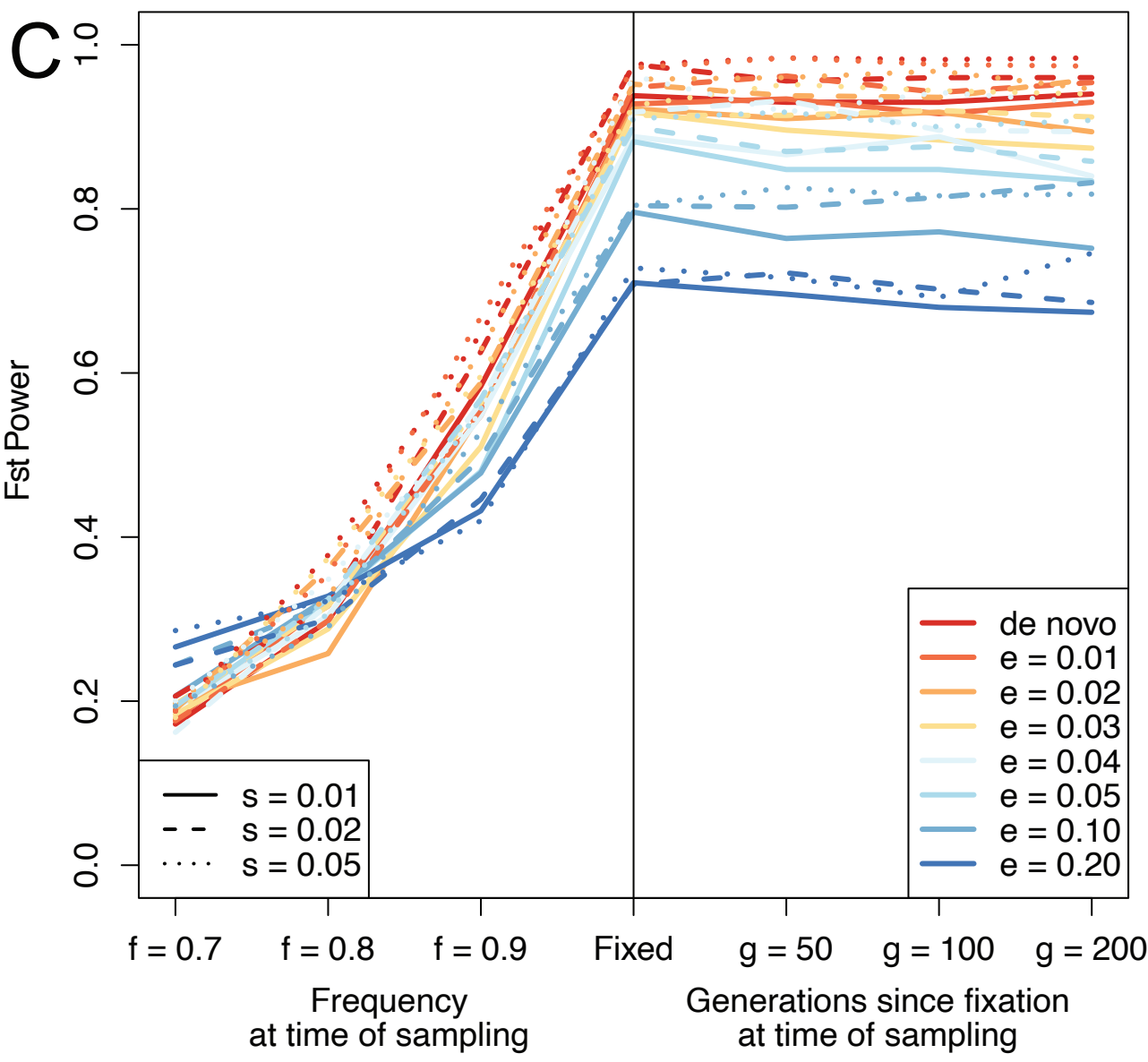

Supplement: Supplementary file 8 — Figure S4. Power curves for (A) nSL, (B) XP‐EHH, and (C) FST. The parameters are e (frequency at which selection begins, e > 0 indicates soft sweep), f (frequency of selected mutation at sampling), g (number of generations since fixation), and s (selection coefficient). [file EVL3-5-408-s009.pdf]

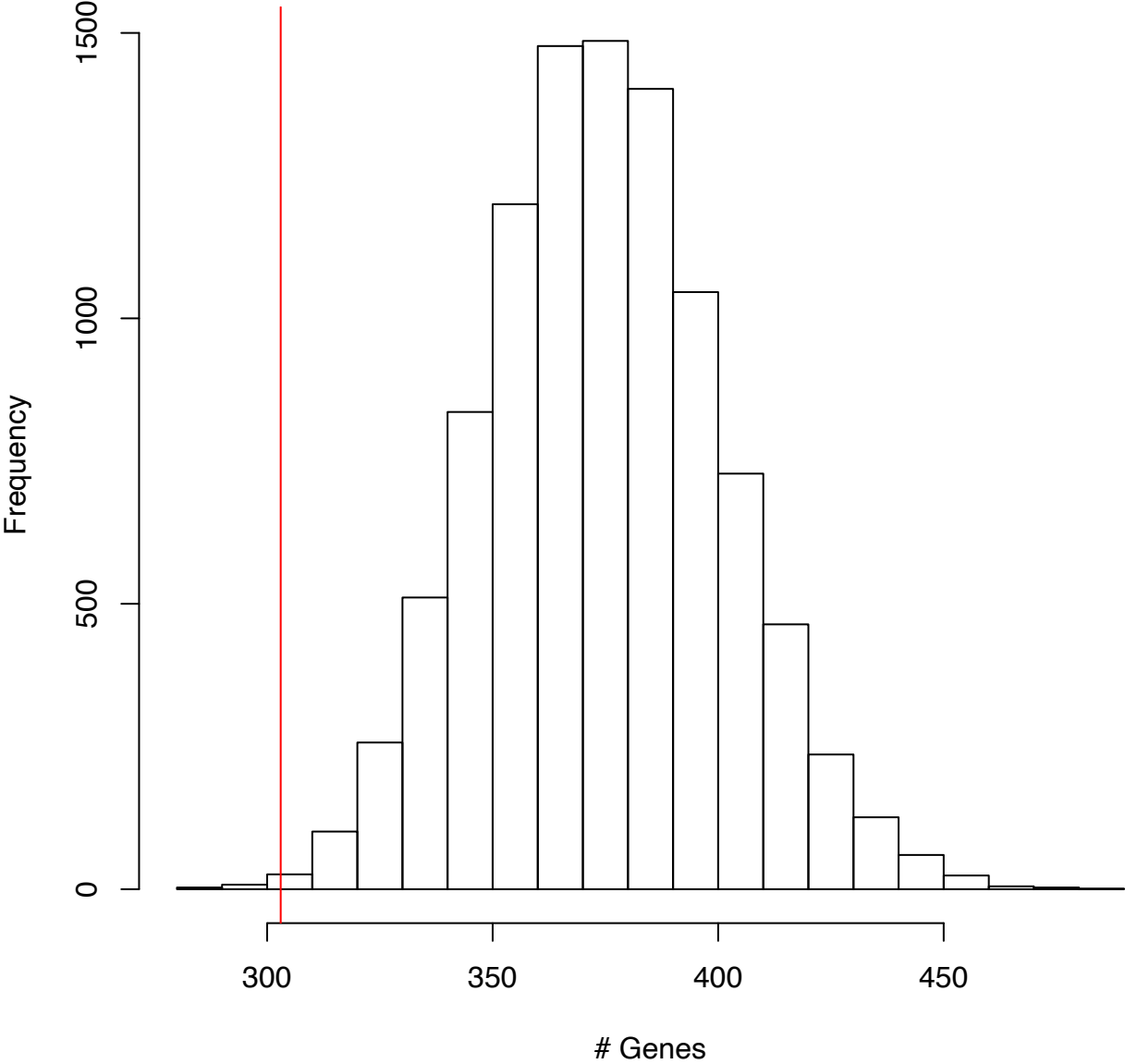

Supplement: Supplementary file 9 — Figure S5. A permutation test (10,000 replicates) that shuffles 270 100kb regions around the macaque genome and counts the number of unique genes overlapping. The red vertical line marks the 303 genes found in the real data analysis. The probability of observing 303 or fewer genes is 1.4×10^−3^, indicating the analysis is not randomly choosing gene regions. [file EVL3-5-408-s002.pdf]

Proportion of scores > 2 in window

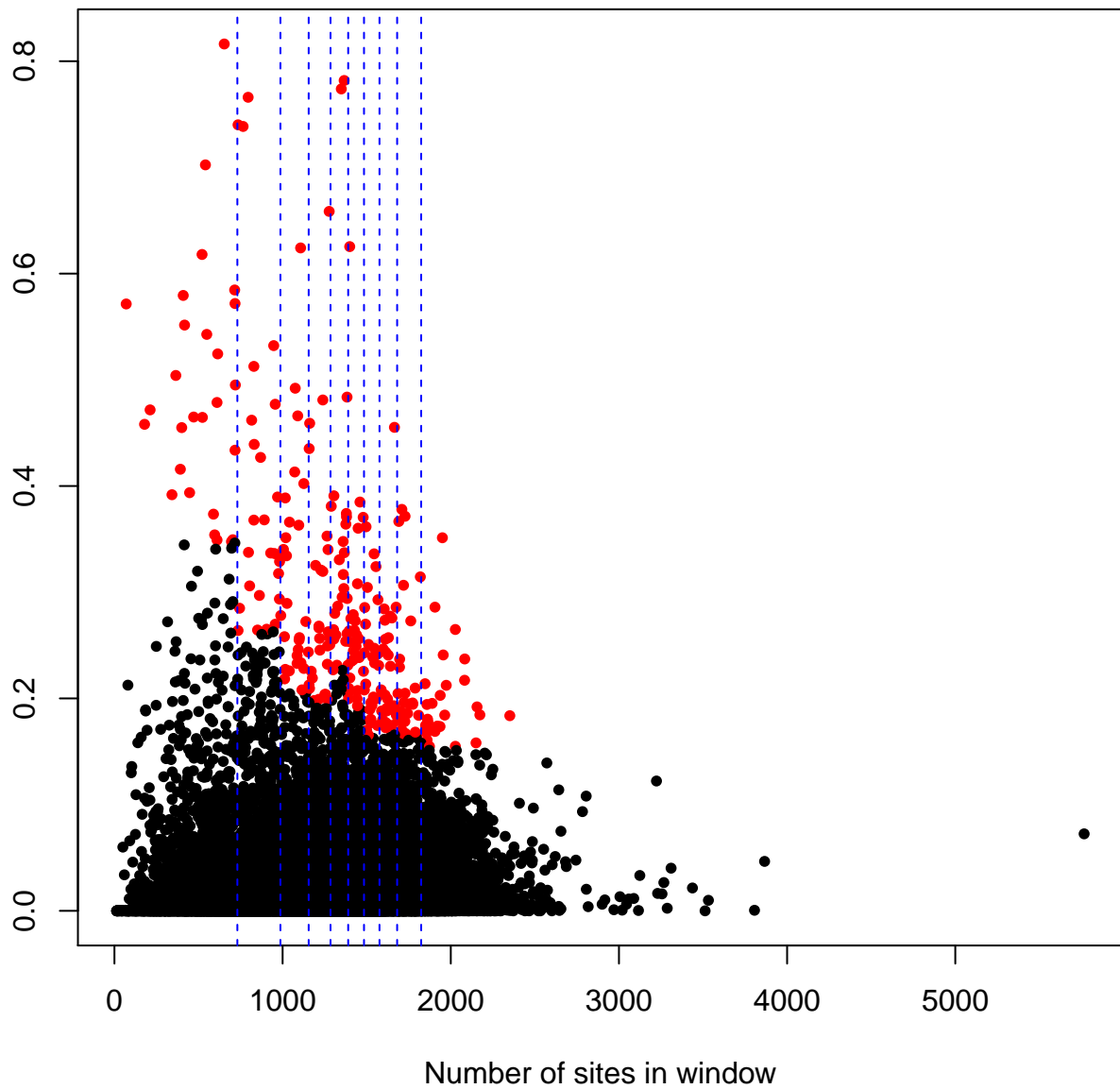

Supplement: Supplementary file 10 — Figure S6. Proportion of scores > 2 versus number of sites in window. Blue vertical dashed lines indicate bin boundaries. Each circle is a window, red dots indicate a proportion of scores > 2 beyond the 1% threshold for that bin. [file EVL3-5-408-s001.pdf]

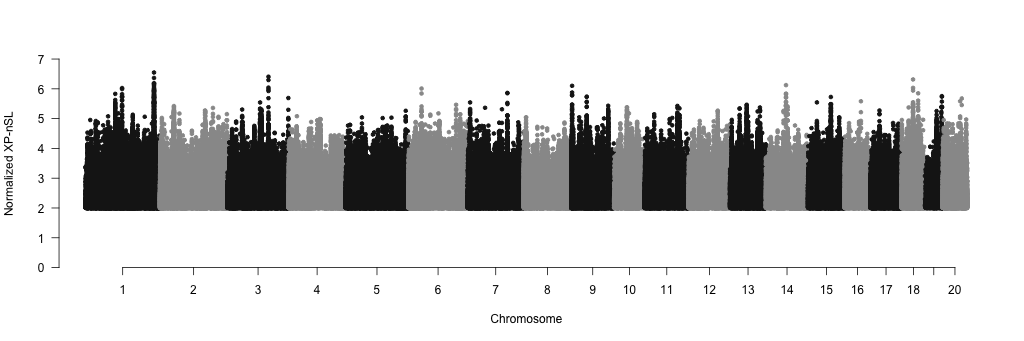

Supplement: Supplementary file 11 — Figure S7. A Manhattan plot of normalized XP‐nSL scores across the genome. Due to a very large number of points, only scores > 2 were plotted. [file EVL3-5-408-s004.tif]
